# Case report and literature review: plexiform schwannoma in the penile and inguinal region in a child

**DOI:** 10.3389/fonc.2024.1356000

**Published:** 2024-03-01

**Authors:** Xiaoying Qi, Yifei Tan, Yanru Feng, Dan Ma, Ling Wang, Houqing Pang

**Affiliations:** ^1^ Department of Ultrasound, West China Second University Hospital, Sichuan University, Chengdu, Sichuan, China; ^2^ Key Laboratory of Birth Defects and Related Diseases of Women and Children, West China Second University Hospital, Sichuan University, Chengdu, Sichuan, China; ^3^ Department of Thoracic Surgery, West China Hospital, Sichuan University, Chengdu, Sichuan, China; ^4^ Department of Ultrasound, Meishan Women and Childrens’ Hospital, Alliance Hospital of West China Second University Hospital, Sichuan University, Meishan, Sichuan, China

**Keywords:** schwannoma, penis, inguinal region, plexiform, children

## Abstract

Penile schwannoma is an uncommonly seen peripheral nerve tumor, of which penile plexiform schwannomas (PS) is extremely rare that has only been reported in several adults. We present a case of penile PS with a similar lesion in inguinal region in a 9-year-old child, which appeared as painless masses and rapidly growing within one year. Penile ultrasonography suggested well-defined lesions with limited vascularity. Both masses presented with low-to-intermediated signal intensity and no definite enhancement in computed tomography. The lesions were completely resected with minimal intraoperative bleeding, and a diagnose of benign PS was confirmed based on H&E staining and positive S-100 expression in immunohistochemistry. There was no evidence of tumor recurrence or metastasis after 6 months of follow-up. Only 6 cases of penile schwannoma in children were recorded, of which 5 were malignant, and none was PS. The malignancy rate of penile schwannoma in children may be overestimated due to delayed diagnose of benign ones. A rapidly growing penile mass with a suspected metastatic lesion in inguinal region could be easily misdiagnosed as malignant. This case report and literature review is expected to assist clinicians in getting a comprehensive understanding of children penile schwannomas and choosing the best management strategy when faced with this rare condition.

## 1 Introduction

Schwannoma, the most common peripheral nerve tumor in adults, is an encapsulated tumor composed of Schwann cells, with an incidence of 0.6 per 100,000 people annually (1). They may occur anywhere in the soft tissue but prefer the head, neck and limbs. Primary tumors of the penis, however, are extremely uncommon, with penile schwannomas even rarer, still. Based on histological features and growth patterns, schwannoma can be classified into the following types (2): classic schwannoma, cellular schwannoma, plexiform schwannoma (PS), ancient schwannoma, epithelioid cell schwannoma, and reticular/microcystic schwannoma. PS is a rare subtype, accounting for approximately 4.3% of schwannomas (3). Previously, only six cases of adult penile PS have been reported (4–9). Herein, we present a case of PS occurring in the penis and inguinal region simultaneously in a child. It is extraordinarily rare. Because of this, when a urologist or radiologist encounters such tumors in clinic, it is often difficult to make an accurate diagnosis. The current report presents a detailed description of the clinical course, imaging examination, surgery and pathology. We also compare the characteristics of penile schwannoma between children and adults based on a comprehensive literature review and summarize the features of penile PS previously reported.

## 2 Case presentation

A previously healthy 9-year-old boy presented with a palpable mass on his penis, which was only the size of a soybean when first discovered one year ago. Subsequently, the mass extended along the dorsal aspect of the entire penis, and another mass was palpable on the left inguinal area six months later. He did not experience any penile pain or urinary obstruction, and no family history of a genetic nerve sheath tumor was reported. Physical examination revealed no café-au-lait spots. A soft subcutaneous mass with moderate mobility, about 5.0 cm in length, was palpable at the base and dorsum of the penis. A similar 2.0 cm nodule appeared in the left inguinal region, without tenderness, but was hardly movable. Blood and urine tests were all within normal range.

**Figure 1:**
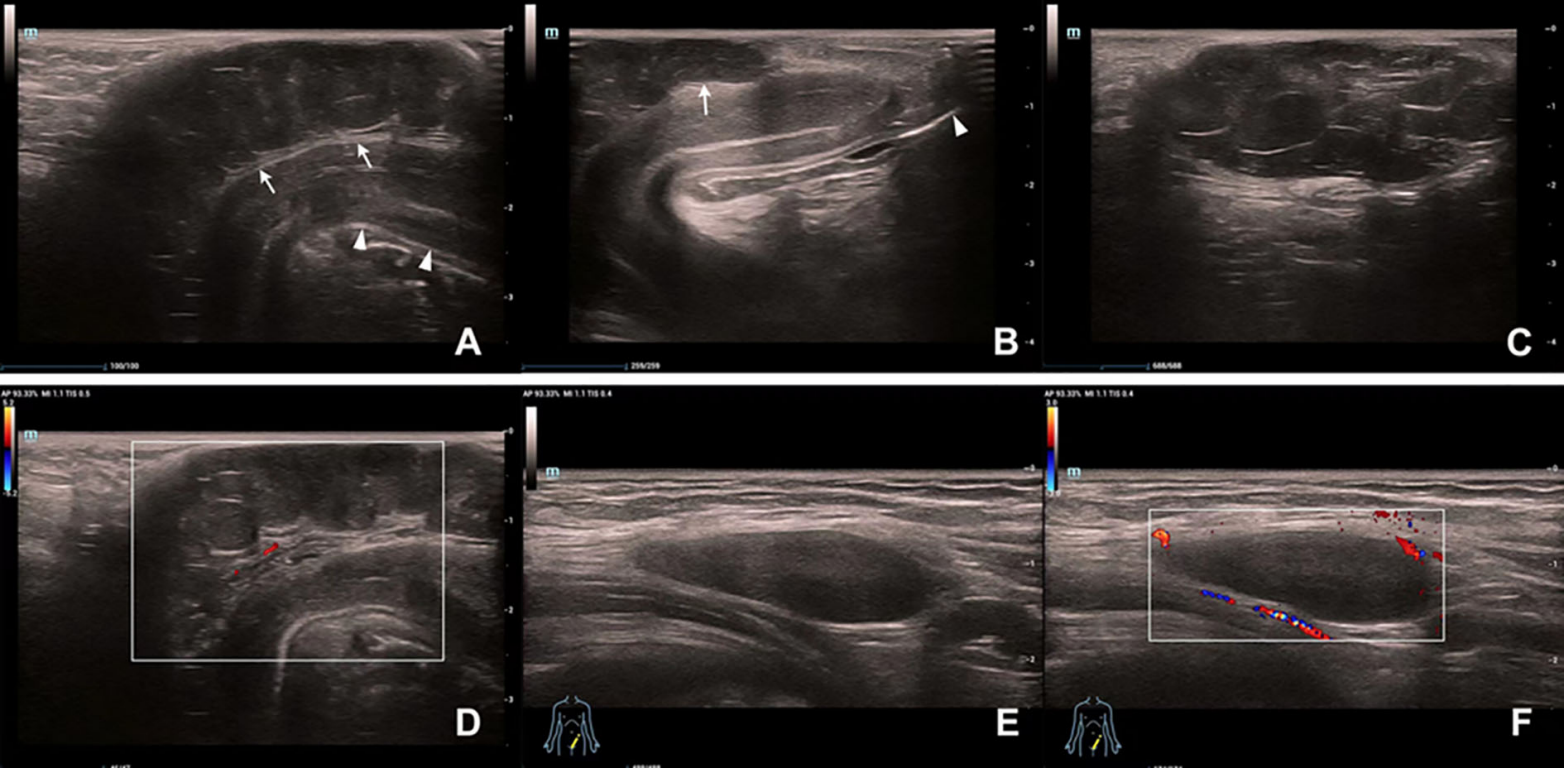
Ultrasound findings of the masses on the penis and in the left groin area. **(A)** A hypoechoic mass (arrow) with a hyperechoic capsule on the dorsal side of the penile shaft (arrowhead). **(B)** The distal part of the mass (arrow) does not involve the glans (arrowhead). **(C)** Posterior acoustic enhancement and multiple linear hyperechoic separations can be seen inside the penile mass. **(D)** Vascularity of the penile mass was not abundant on color Doppler flow evaluation. **(E)** A homogeneous hypoechoic mass in the groin area. **(F)** The inguinal mass showed hypovascularity on Color Doppler ultrasonography.

**Figure 2:**
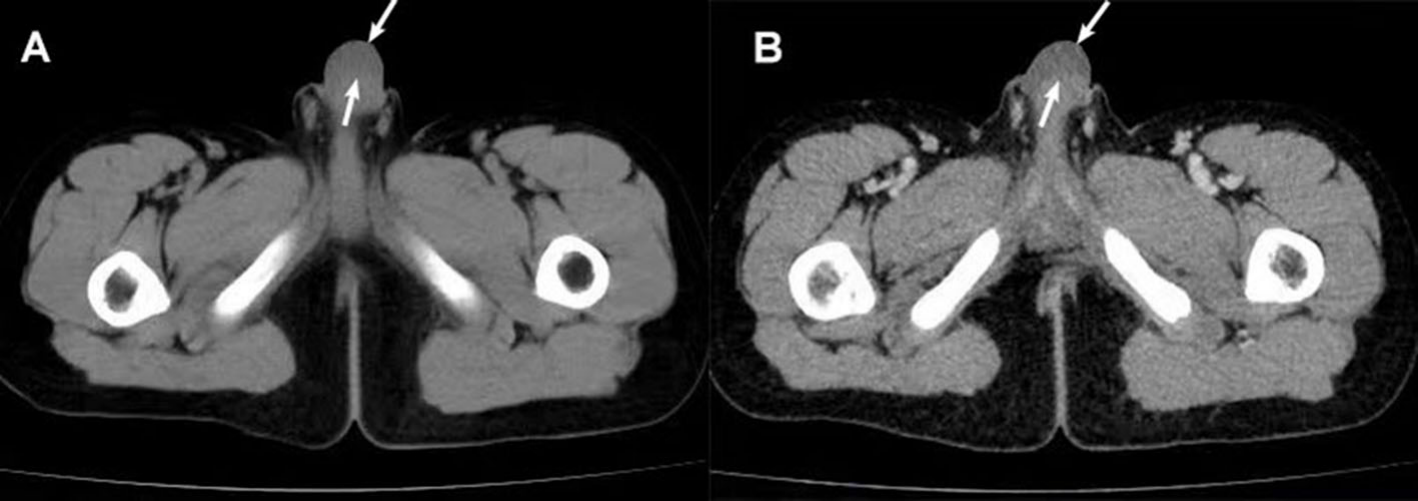
CT scan findings of the penile mass. **(A)** CT plain scan: a uniform low-density mass (arrow) was seen under the penile skin. **(B)** Enhanced CT: No definite enhancement of the mass (arrow) after enhancement.

Penile ultrasonography suggested a hypoechoic mass extending from the base to the dorsal side of the penis and measured 5.2 x 1.1 x 3.5 cm. The mass showed a clear boundary distinguishing it from the penile corpus cavernosum, with internal linear echogenic septa, slightly enhanced posterior echogenicity, and very limited vascularity inside. A well-defined homogeneous hypoechoic mass without notable septa was found in the left groin area (**Figure 1**). Both masses presented with low-to-intermediated signal intensity in further evaluation with pelvic computed tomography (CT). No definite enhancement was displayed on postcontrast images (**Figure 2**).

**Figure 3:**
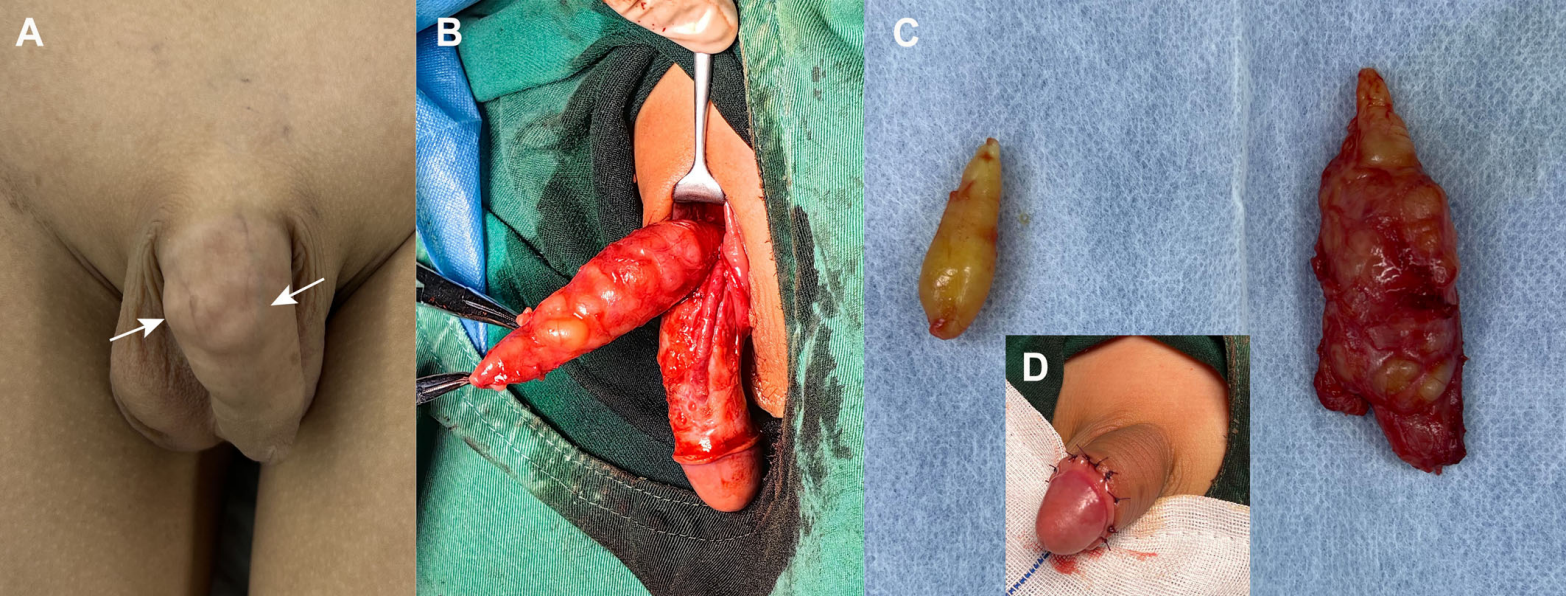
Intraoperative imagings of penile and inguinal schwannomas. **(A)** Grossly, palpable tissue mass was observed on the dorsum of the penis, near the base of the shaft, with a bumpy, multi-nodular appearance (arrow). **(B)** Intraoperative findings suggested that the yellow mass between the deep and superficial fascia on the dorsal side of the penis was multi-nodular and demarcated from the corpus cavernosum. **(C)** Final gross specimens of the penile mass (right) and inguinal mass (left) were all yellow nodular. **(D)** Postoperatively, the appearance of the penis was less affected.

Given the size of the tumor and its increasing trend, a surgical removal was planned. Resection of both masses was performed under general anesthesia. The penile mass was between deep and superficial fasciae, with intact capsule and clear margins, which made it easy to separate the mass from adjacent tissue. Beneath the left inguinal ligament, a similar mass was detected on the outer side of the femoral artery. Both lesions were spindle-shaped with yellowish surface and were completely resected with minimal intraoperative bleeding. Intraoperative frozen section pathological examination indicated a benign neural-origin tumor. Thus, no extended excision was performed. The appearance of the penis was not notably impacted postoperatively (**Figure 3**).

**Figure 4:**
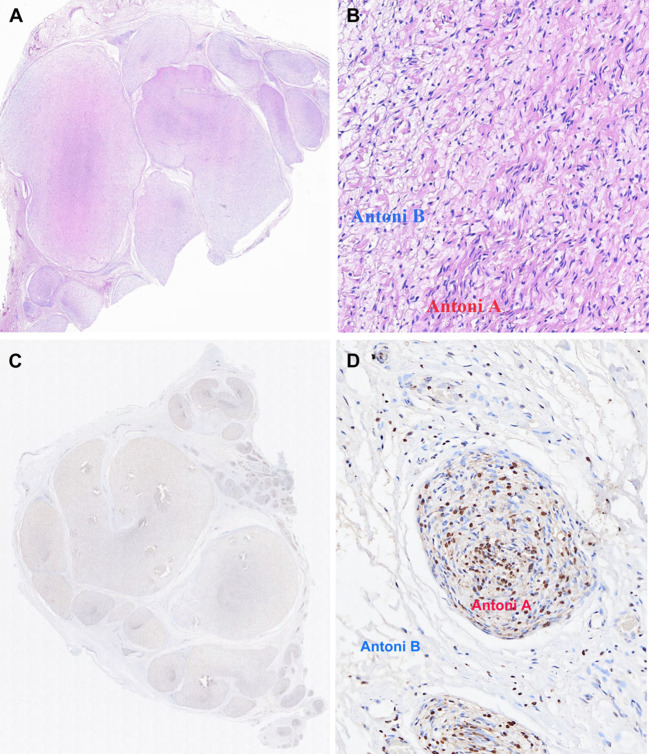
Final pathology of penile mass specimen excised during operation. Hematoxylin-eosin (H&E) stain: **(A)** The tumor was divided into multiple nodules by fibrous tissue (HE,×10). **(B)** Hypercellular Antoni A areas: compact spindle cells with no atypia or mitoses arranged in bundles or interlacing fascicles. Hypocellular Antoni B areas: spindle cells arranged haphazardly in a loose stroma. (HE, ×200). Immunohistochemistry stain: **(C)** Diffuse expression of S100. **(D)** S100 expression primarily concentrated in the nucleus of Antoni A areas.

Hematoxylin-eosin (H&E) staining (**Figures 4A, B**) showed that fibrous tissue divided the tumor into multiple nodules. A typical pattern of mixed Antoni A and Antoni B areas was demonstrated. The predominant distribution was Antoni A areas, characterized by spindle-shaped cells with palisading nuclei arranged in a fascicular or crisscross pattern. Immunohistochemistry staining demonstrated diffuse expression of S-100 (**Figure 4C**), mainly in Antoni A areas and primarily concentrated in the nucleus (**Figure 4D**). Both the penile and inguinal masses were diagnosed as plexiform schwannomas. There was no evidence of tumor recurrence or metastasis after 6 months of follow-up. The patient urinated well and the erectile function of penis was normal.

## 3 Discussion

While the penis and perineum have abundant nerve innervation, schwannoma of penis is a rare condition, with only 47 cases reported. The majority of penile schwannomas are benign, slow-growing neoplasms, and the average patient age was 39.2 years (10). Malignant transformation of these cells is uncommon and only occurs in 9 cases. Penile schwannoma generally presents as a solitary and painless tumor that is most commonly on the dorsal shaft in children. While in adults, these tumors have caused pain, curvature erectile, sexual dysfunction and urinary tract obstruction (1).

The incidence, growth rate and risk of malignant transformation may differ between pediatric and adult penile schwannomas. **Table 1** shows the clinical characteristics of penile schwannomas in adults and children respectively. Penile schwannomas in children were all solitary mass with a shorter duration of illness (1 week-2 years) and a faster growth rate. In 6 children cases, 5 (83%) were diagnosed as malignant, of which 2 were associated with NF1 and 2 underwent malignant transformation from the plexiform neurofibroma (PNF). Only 1 with a malignant lesion developed a recurrence, and no distant metastases were observed. Most of the 41 adult cases had a long course (2 months-25 years) and presented with a single nodule (73.2%). 4 cases (9.8%) were graded as malignant, one of which was associated with NF1. One malignant case presented distant metastasis, and recurrence was observed in 3 benign and 2 malignant cases. Notably, the penile mass may have been present since childhood in some adult cases (4, 5, 11, 12). Delay in seeking medical assistance because of fear or shame of lesion of private parts, tolerable symptoms, lack of medical resources or health education, or tumor excision without pathological proof may result in underestimated incidence and overestimated malignancy rate of penile schwannoma in children.

**Table 1:** Comparison of penile schwannoma between children and adults.

Clinical features		Children	Adult
Number of people		6	41
Age (years old)		1.2-16	19-75
Median age(years old)		9	39
Average age(years old)		8.86 ± 6.83	42.56 ± 16.40
Symptom duration		1week-2 years	2months-25 years
Clinical symptoms	Pain/discomfort	–	13
	Sexual dysfunction	–	8
	Urinary symptoms	–	2
Pathological classification	Benign	1	37
	Malignant	5	4
NF1		2	1
NF2		–	–
SWN		–	1
PS		–	6
PNF		2	–
Tumor size(cm)		1.3-8	0.5-20
Number of tumors	Single	6	30
	Multiple	–	11
Necrosis		0	2
Location	Dorsal	3	22
	Ventral	–	5
	Left/right side	–	3
	Glans/prepuce	1	10
	Root/scrotum	2	–
	Frenulum	–	1
Operation	Tumor resection only	5	38
	Total or partial penectomy	1	3
Recurrence	Benign	–	3
	Malignant	1	2
Metastasis		–	1

NF1, neurofibromatosis type 1; NF2, neurofibromatosis type 2; SWN, schwannomatosis; PS, plexiform schwannoma; PNF, plexiform neurofibroma.

PS represents a rare variant of schwannoma and is characterized by a plexiform or multinodular growth pattern (6). Only 6 cases of penile PS, all adults, were recorded. (**Table 2**). The median age was 30 years (21-75 years), and the median mass size was 1.75 cm (1.1-6.0 cm). The most prevalent site is the penile shaft, which was found in 4 cases (66.7%) and all experienced varying degrees of pain or discomfort. In the remaining 2 cases, tumors appeared on the glans without apparent symptoms. Except for one case of glans penis, the course of other diseases is relatively short (1-5 years). PS is previously unreported in the penis in the pediatric age group. Our case delineates the first of which to occur in a patient this young with a tumor size greater than 5.0 cm in the penis. This lump rapidly grew to over 5cm within one year, accompanied by a similar lesion in the inguinal region, which could be easily misdiagnosed as a malignant tumor with inguinal metastasis.

**Table 2:** Features of penile plexiform schwannoma.

Reference	Age	Site	Duration	Number	Chief complain	Size (cm)	Texture	Histologic finding	Immuno-histochemistry
**Fletcher et al.1986 (** 4)	21	Root	Many years	1	Tenderness	1.5	–	Moderate pleomorphism, no mitosis, entire Antoni A areas, occasional Verocay bodies	–
**Ghaly et al.2000 (** 5)	36	Glans	20 years	1	Slowly enlarging	2.5	–	Haemosiderin deposition and hyalinized blood vessels	–
**Gupta et al.2005 (** 6)	75	Shaft(dorsum)	1year	1	Increased in size; urinary frequency an-d hesitation	5	Firm	Mild pleomorphism,no mitosis, entire Antoni A	S-100(+)
**Lin et al.** **2010 (** 7)	44	Glans	1 year	1	Slowly growing	1.1	Elastic	Mainly Antoni A areas, interspersed Antoni B areas	S-100(+)
**Pan et al.** **2012 (** 8)	35	Shaft(left side)	5 years	1	Increased in size; penis curvature; intercourse trouble	6	–	–	–
**Gkekas et al.2019 (** 9)	39	Shaft(dorsum)	2 years	2	Penis deviation on erection;inte-rcourse pain	2	Soft	Antoni A and B areas, nuclear palisading	S-100(+)

Imaging studies can assist in diagnosing penile schwannomas, although their specificity is limited. Ultrasonography usually shows a well-demarcated and uniform hypoechoic mass with posterior acoustic enhancement (13). Cystic or necrotic degeneration is rarely seen (14). Hypervascularity may occur in 38% of penile schwannomas (15). Although without increased vascularity, the lesions did appear multiple internal hyperechoic lines in our case, which have never been reported. CT scans often reveal homogeneous low-density nodules with clear, smooth borders and a slightly higher-density peripheral capsule. MRI features include elliptical tumors with eccentric growth along nerve trunks, appearing as low to moderate signals on T1-weighted images and high signals on T2-weighted images (13). Preoperative fine-needle aspiration biopsy can preliminarily determine whether the mass is malignant, and sharp radiating pain during the biopsy can serve as a crucial clue to a nerve-derived tumor (6).

Typical schwannoma is characterized by a single nodule enveloped by a fibrous capsule. The nodule is mainly composed of spindle-shaped cells, often palisading, with densely cellular zone of Antoni A areas, and hypocellular Antoni B areas consisting of sparse spindle cells in myxoid stroma. PS presents as multiple nodules primarily composed of Antoni A areas. Immunohistochemically, they show similar diffusely strong expression of the neural marker protein S100. Penile PS in adults often demonstrates mild to moderate pleomorphism. In other locations, PS has abundant cell numbers, necrosis, increased nuclear pleomorphism, high mitotic activity and the possibility of local recurrence in children, but without metastatic potential (3). The higher mitotic activity and increased cellularity may be responsible for the rapid increase in mass size. Notably, the penile PS in the current report showed predominant nuclear distribution that differs from the diffuse and uniform expression of S100 in the cytoplasm and nucleus observed in some adult cases (1, 16). This disparity may be attributed to the variance between penile PS in children and adults, however, additional case studies are required to validate this conjecture.

Schwannomas are primarily isolated and sporadic tumors, while multifocality may be associated with schwannomatosis. Inactivating mutations in tumor suppressor genes, such as SMARCB1, LZTR1 and NF2, often give rise to schwannomatosis. A loss of function in the SMARCB1 and LZTR1 gene is also a causative factor in certain childhood tumors and syndromes that may affect intelligence, such as atypical teratoid/rhabdoid tumors, Coffin-Siris syndrome and Noonan syndrome (17). Out of 87 cases of head and neck PS reported by Berg JC, 9 were associated with a syndrome, of which 4 were children (3). Therefore, genetic syndromes or certain specific tumors should be considered promptly in children with multiple schwannomas or PS. There have been no reports of childhood penile PS. It is currently unclear whether penile PS behaves biologically like the head and neck PS.

PS should be identified with PNF. PNF is closely related to NF1 and has a 2%-5% risk of malignant transformation, which increases to 10%-30% when symptoms are present (17). The tumors consist of Schwann cells, fibroblasts, and perineural cells, without capsules or typical Antoni A/B regions. Immunohistochemistry shows weak positivity for S100. Furthermore, unlike penile PS, the aggressive growth of PNF can lead to arterial steal from the corpus cavernosum, directly causing erectile dysfunction (18).

Penile schwannomas are well-encapsulated lesions that generally attach eccentrically to nerves. Thus, they are easily removable without any appreciable nerve insult (16). PS have a higher local recurrence rate, possibly due to irregular and multifocal growth patterns, making it challenging to define clear tumor margins during excision, leading to inadequate removal (6). Close follow-up after resection is necessary to monitor penile functional recovery and tumor recurrence. Only 4 of 47 cases underwent total or partial penectomy. One case involved a 14-month-old child with a preoperative diagnosis of malignant schwannoma. The patient underwent complete penectomy, partial ureterectomy, perineal urethrostomy, partial scrotectomy, and testicular relocation, followed by radiation. Though the child survived, complex urogenital reconstruction and penile transplantation were required (19). When dealing with penile tumors in children, the decision to perform complete or partial penile excision should be made judiciously to avoid overtreatment. In cases of uncertainty, intraoperative frozen section biopsies can help determine the appropriate surgical extent.

This case report summarizes the features of penile PS that are difficult to detect in clinical practice and compares the characteristics of penile schwannoma between children and adults. However, it is based on a single case, which may not be representative, thereby limiting its clinical applicability. Collecting and summarizing more case series would provide a more comprehensive and systematic understanding of penile PS in children, benefiting a larger number of patients. Besides, the follow-up period was relatively short. We will continue to track and follow up, and if necessary, we will provide follow-up reports.

## 4 Conclusion

Penile schwannomas in children are rare and preoperative diagnosis can be challenging. We present the first case of pediatric penile PS accompanied by a similar lesion in the inguinal region, which was completely removed without causing nerve damage. Although the rate of malignancy may be overestimated, it should be noted that penile schwannomas in children may differ from those in adults, as they may grow faster and be bound up with genetic syndromes or certain specific tumors that affect intelligence. Care also should be taken to differentiate them from PNF, as the former is benign while the latter carries a high risk of malignant transformation. Comprehensive understanding of penile schwannomas in children is essential to make the correct diagnosis and determine the optimal management approach.

## Data availability statement

The original contributions presented in the study are included in the article/supplementary material. Further inquiries can be directed to the corresponding author.

## Ethics statement

Written informed consent was obtained from the individual(s), and minor(s)’ legal guardian/next of kin, for the publication of any potentially identifiable images or data included in this article.

## Author contributions

XQ: Writing – original draft, Writing – review & editing. YT: Writing – original draft. YF: Writing – original draft. DM: Writing – original draft, Formal analysis. LW: Writing – original draft, Data curation. HP: Writing – original draft, Writing – review & editing.

## References

[1] Kim SH Ahn H Kim KH Kim DS Yang HJ. Penile schwannoma mistaken for hemangioma: a rare case report and literature review. Transl Androl Urol. (2021) 10:2512–20. doi: 10.21037/tau-21-239 PMC8261408 34295737

[2] Magro G Broggi G Angelico G Puzzo L Vecchio GM Virzì V. Practical approach to histological diagnosis of peripheral nerve sheath tumors: an update. Diagnostics. (2022) 12:(6):1463. doi: 10.3390/diagnostics12061463 35741273 PMC9222088

[3] Berg JC Scheithauer BW Spinner RJ Allen CM Koutlas IG. Plexiform schwannoma: a clinicopathologic overview with emphasis on the head and neck region. Hum Pathol. (2008) 39:633–40. doi: 10.1016/j.humpath.2007.10.029 18439936

[4] Fletcher CDM Davies SE. Benign plexiform (multinodular) schwannoma: a rare tumour unassociated with neurofibromatosis. Histopathology. (1986) 10:971–80. doi: 10.1111/j.1365-2559.1986.tb02595.x 3096870

[5] Ghaly AF Orange GV. Not every penile lump is a wart! Schwannoma of the penis. Int J STD AIDS. (2000) 11:199–200. doi: 10.1258/0956462001915543 10726948

[6] Gupta SK Singh S Kumar CP Marwah N Gupta A. Fine needle aspiration cytology of a non-ulcerated penile lump. Cytopathology. (2005) 16:312–4. doi: 10.1111/j.1365-2303.2005.00231.x 16303046

[7] Lin TC Wu PY Lin TY Lee TL. An infrequent plexiform variant of schwannoma of the glans penis: a rare finding. Asian J Androl. (2010) 12:455–7. doi: 10.1038/aja.2009.96 PMC3739260 20118951

[8] Pan F Li B Kunwar KJ Zhang Q Xiao Y Zeng F. Neuroimage: giant plexiform schwannoma of the penis. Eur Neurol. (2013) 69:118–8. doi: 10.1159/000342238 23207604

[9] Gkekas C Kalyvas V Symeonidis EN Malioris A Papathanasiou M Kalinderis N. Plexiform schwannoma of the penis: A rare subtype of genital schwannoma. Case Rep Urol. (2019) 2019:1–4. doi: 10.1155/2019/1752314 PMC6470439 31073418

[10] Nguyen AH Smith ML Maranda EL Punnen S. Clinical features and treatment of penile schwannoma: a systematic review. Clin Genitourin. Cancer. (2016) 14:198–202. doi: 10.1016/j.clgc.2015.12.018 26797586

[11] Chan WP Chiang SS Huang AH Lin CN. Penile frenulum neurilemoma: A rare and unusual genitourinary tract tumor. J Urol. (1990) 144:136–7. doi: 10.1016/S0022-5347(17)39394-1 2359163

[12] Kumar GP Sukumar S Bhat SH Nambiar A. Schwannoma of the penis: A common tumour at a rare site. Scand. J Urol Nephrol. (2006) 40:166–7. doi: 10.1080/00365590500499776 16608817

[13] Song Z Zhang Z Xu S Qiu Y Ouyang J. Multiple penile schwannomas. Urol Case Rep. (2022) 43:102107. doi: 10.1016/j.eucr.2022.102107 35586399 PMC9109180

[14] Lee CH Wu CJ Chen YL Huang GS Tang SH. Multiple penile schwannomas and their magnetic resonance imaging characteristics. J Androl. (2012) 33:167–9. doi: 10.2164/jandrol.111.012997 21636734

[15] Huang LC Wang HZ Chu YC Ng KF Chuang CK. Clinicopathological presentation and management of penile schwannoma. Sexual Med Rev. (2020) 8:615–21. doi: 10.1016/j.sxmr.2019.12.001 31926907

[16] Mejri R Dali KM KaysChaker Mokhtar B Ben Rhouma S Nouira Y. Isolated penile schwannoma: A rare case report. Urol Case Rep. (2022) 40:101866. doi: 10.1016/j.eucr.2021.101866 34646741 PMC8497845

[17] Schraepen C Donkersloot P Duyvendak W Plazier M Put E Roosen G. What to know about schwannomatosis: a literature review. Br J Neurosurg. (2022) 36:171–4. doi: 10.1080/02688697.2020.1836323 33263426

[18] Issa B Mansour E Jabbour G Chikhani C Mansour H Jabbour M. Plexiform penile neurofibroma: A case report of a rare entity in a pre-pubertal child. Urology. (2021) 156:124–6. doi: 10.1016/j.urology.2021.06.001 34129894

[19] Agard H Parekh N Clark C Massanyi E Murthy A McMahon D. Intermediate follow-up and management of previously reported Malignant peripheral nerve sheath tumor of the penis. Urology. (2020) 135:133–5. doi: 10.1016/j.urology.2019.09.028 31586472

